# “Jing-Ning Granules” Can Alleviate Attention Deficit Hyperactivity Disorder in Rats by Modulating Dopaminergic D2/D1-Like Receptor-Mediated Signaling Pathways

**DOI:** 10.1155/2022/9139841

**Published:** 2022-10-28

**Authors:** Jie Ding, Yiyun Ding, Jingjing Wu, Jialin Deng, Qingyang Yu, Junhong Wang

**Affiliations:** ^1^Department of Pediatrics, Dongzhimen Hospital, Beijing University of Chinese Medicine, Beijing 100700, China; ^2^School of Psychology, Capital Normal University, Beijing 100048, China; ^3^Department of Pediatrics, The First Affiliated Hospital of Henan University of Chinese Medicine, Zhengzhou 450000, China; ^4^Department of Pediatrics, Beijing Huaxin Hospital, The First Affiliated Hospital of Tsinghua University, Beijing 100016, China; ^5^Department of TCM, Children's Hospital of Chongqing Medical University, Chongqing 400016, China

## Abstract

**Background:**

Attention deficit hyperactivity disorder (ADHD) is a neurodevelopmental disorder characterized by attention deficit, hyperactivity, and impulsivity. Jing-Ning Granules (JNG) is a traditional Chinese medicine (TCM) that can alleviate ADHD. Although JNG is commonly used for the effective treatment of ADHD and has obtained the national invention patent, the exact mechanism of action remains unclear.

**Objective:**

In this study, we examined the effect and mechanism of JNG in spontaneously hypertensive rats (SHRs). We hypothesized that JNG affects dopaminergic D2/D1-like receptors and related pathways.

**Materials and Methods:**

Six rat groups were used in the experiment: Wistar-Kyoto rats (WKY, control group) and five SHR groups, including a model group; atomoxetine (ATX, positive control) group; and low, medium, and high-dose JNG groups. The corresponding treatments were daily administered to each group for 6 weeks. A behavioral test, including a step-down test and open field test (OFT), was carried out at the end of treatment. After the behavioral test, all animals were sacrificed, and the brain tissue was collected and analyzed ex vivo; histopathological analysis was performed to assess the pathological changes of the hippocampus; expression of D1-like and D2-like receptors, sensor protein calmodulin (CaM), protein kinase A (PKA), and calcium/calmodulin-dependent serine/threonine protein kinase (CaMKII) in the striatum and hippocampus was measured by western blot and real-time quantitative PCR (RT-PCR); cyclic adenosine monophosphate (cAMP) levels in the striatum were analyzed using an enzyme-linked immunosorbent assay (ELISA), while the level of Ca^2+^ in the striatum was analyzed by a calcium kit.

**Results:**

Our results showed that ATX or JNG could ameliorate the hyperactive/impulsive behavior and cognitive function of ADHD by promoting neuroprotection. Mechanistically, ATX or JNG could prompt the expressions of Dl-like and D2-like receptors and improve the mRNA and protein levels of cAMP/PKA and Ca^2+^/CAM/CAMKII signaling pathways.

**Conclusion:**

These results indicate that JNG can produce therapeutic effects by regulating the balance of D2/D1-like receptor-mediated cAMP/PKA and Ca^2+^/CaM/CaMKII signaling pathways.

## 1. Introduction

Attention deficit hyperactivity disorder (ADHD) is a neurodevelopmental disorder that begins in childhood and is characterized by attention deficit, hyperactivity, and impulsivity that are not in line with the developmental levels [1, 2]. Different assessment methods and diagnostic criteria are used for ADHD; yet, there is no simple test to determine ADHD. The incidence rate of ADHD is high, especially among minors (5%) [3, 4]. In addition, ADHD often presents with one or more several psychiatric comorbidities, such as autism spectrum disorder (ASD), reading disability, anxiety disorders, and oppositional defiant disorder, which remain the most prevalent disturbances across the lifespan [5, 6]. While it often appears in childhood, some cases were reported in adolescence and adulthood, which further challenges the diagnosis. Low employment rates, low income, and high crime rates have been associated with ADHD in the adult stage [7, 8]. Thus, the disease has an extensive and persistent or even lifelong impact on patients' families, education, social life, and other aspects, causing serious social and economic burdens.

**Figure 1:**
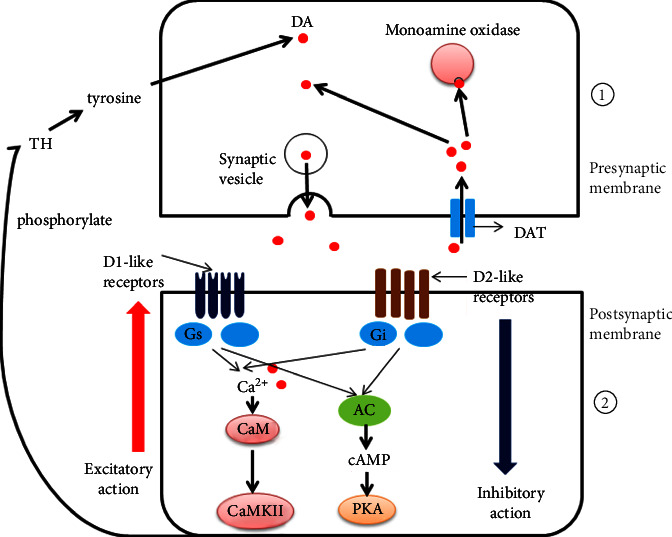
A schematic plot of DA transport in the extracellular and intracellular pathways. ① DA is released to the synaptic cleft by vesicular exocytosis, acting on DA receptors on the postsynaptic membrane. DA is then reuptaken by dopamine transporters (DAT) back into neurons, partly reabsorbed, and utilized and partly inactivated by inactivating enzymes (MAO). ② DA in the synaptic cleft acts on D1-like and D2-like receptors, which exert different physiological functions. The Dl-like receptors are coupled to Gs/olf proteins that activate the cAMP/PKA pathway, whereas the D2-like receptors are coupled to Gi/o proteins that inhibit AC activity and eventually lead to reduced PKA levels; similar effects are observed on the Ca^2+^/CAM/CAMKII pathway.

The onset of ADHD is affected by complex multifactor, such as heredity, neurobiochemistry, and environment. [9]. While the etiology and pathogenic mechanisms underlying ADHD remain largely elusive, it has been confirmed that dopamine (DA), norepinephrine (NE), and other monoamine neurotransmitters are related to the pathogenesis of ADHD [10, 11], but the specific mechanism is still unclear. Some scholars have put forward the hypothesis of “excitability and inhibitory transmitter imbalance” [12, 13]. DA has two types of receptors: the D1-like receptor (D1R) family (Drd1 and Drd5) and the D2-like receptor (D2R) family (Drd2, Drd3, and Drd4). These receptors mediate all physiological functions of dopamine, such as autonomous activity, sleep regulation, emotion, attention, and cognitive functions [14, 15]. Dopamine receptors are definite targets in the clinical pharmacology of many diseases, such as schizophrenia, Parkinson's disease, ADHD, and Tic disorders [16]. Numerous advances have occurred in understanding that the postsynaptic membrane D1/D2 receptors can lead to the occurrence of ADHD [17, 18]. Thus, targeting this process may be an effective strategy for the clinical prevention and treatment of ADHD. Dopamine receptor functions have typically been associated with the regulation of downstream molecules via G protein-mediated signaling (Figure 1). Recent progress in understanding the complex biology of dopamine receptor-related signal transduction mechanisms has revealed that dopamine-related pathologic conditions may involve a transition from approaches that directly affect receptor function to precise targeting of postreceptor intracellular signaling [15, 19], which means that the therapeutic targets will shift from antagonizing dopamine receptors to regulating expressions of the receptors and their primary action on downstream molecules in the cAMP/PKA and Ca^2+^/CAM/CAMKII signaling pathways.

Atomoxetine (ATX) is the first non-stimulant, selective norepinephrine transporter inhibitor approved by the Food and Drug Administration of the USA for the treatment of ADHD [20, 21]. It has been shown to be efficient in ADHD patients of different ages, genders, and subtypes (inattentive, hyperactive-impulsive, and combined inattentive/hyperactive-impulsive) and commonly used to improve children's cognitive and executive function [22]. However, due to this disease being lifelong, the use of medications is long term [23]. Furthermore, recent studies have reported certain adverse events resulting from ATX exposure, such as decreased appetite, nausea, headache, dry mouth, and liver injury [24]. All issues of drug use and long-term administration will bring out the worries of parents and caregivers about the safety and effectiveness of medications. Therefore, there is an urgency to accelerate alternative possible therapeutic strategies for ADHD.

As an alternative and complementary medicine for the treatment of ADHD, traditional Chinese medicine (TCM) has a unique advantage for the prevention and treatment of ADHD due to its efficacy and safety [25–27]. Jing-Ning Granules (JNG), an effective traditional Chinese herbal formula summarized based on years of clinical experience in the treatment of ADHD and has been awarded a national invention patent (Patent No. ZL201510303541.6), consists of *Pseudostellaria heterophylla* (Miq.), *Rehmannia glutinosa* (Gaertn.) DC, *Schisandra chinensis* (Turcz.) Baill, *Lycium barbarum* L, *Poria cocos* (Schw.) Wolf, *Polygala tenuifolia* Willd, and *Acorus tatarinowii* Schott with the weight ratio of 2 : 2 : 1 : 1 : 2 : 1 : 1, whose active components have been identified by a sensitive and reliable LC-MS/MS method [28]. Previous clinical studies have found that JNG could effectively improve the clinical core symptoms of restlessness, inattention, and low learning efficiency in children with ADHD, with a total effective rate of 85.71%. In addition, JNG has shown better clinical efficacy and safety than orthodox drugs used for treating children with ADHD [29, 30]. Mechanistically, previous experimental studies have shown that JNG can regulate the balance and receptors of neurotransmitters such as DA, NE, and 5-hydroxytryptamine (5-TH) and improve learning, memory, and cognitive functions [31–34]. However, it is not clear whether JNG can regulate the dopaminergic D2/D1-like receptors. In this study, we used spontaneously hypertensive rat (SHR) as model and provided evidence on this topic, by focusing on dopaminergic D2/D1-like receptors and their downstream molecules in the cAMP/PKA and Ca^2+^/CAM/CAMKII signaling pathways, which can provide a greater scientific basis for its application in clinical treatment.

## 2. Materials and Methods

### 2.1. Drugs and Reagents

The Atomoxetine capsules (ATX) were obtained from LILLY Pharmaceutical Co, Ltd. (LILLY, America; Import permit number H20110150). The rat cAMP ELISA kit was purchased from the manufacturer: BG, Batch No: 20170702. The protein lysis buffer for western blot detection was acquired from Ukzybiotech Ltd. (Beijing, China). Required reagents for RT-PCR, including TIANScript RT Kit, SYBR FAST qPCR Kit Master Mix (2×) Universal (US KAPA Biosystems), and Trizol—Invitrogen, chloroform, and isopropanol—were purchased from Beijing Chemical Reagent Factory (Beijing, China).

### 2.2. Source of Plant Samples

JNG includes 7 different Chinese medicinal herbs (Table 1), purchased from Beijing Kang Ren Tang Pharmaceutical Co. Ltd. The granules were dissolved in 50 mL of distilled water and stored at 2°C–8°C.

JNG was uniformly produced by Beijing Kangrentang Pharmaceutical Co., Ltd. (Batch number: 15020671) and purchased from the TCM pharmacy of Dongzhimen Hospital of Beijing University of Chinese Medicine. The quality of the JNG was controlled according to the 2010 edition guidelines of the Pharmacopoeia of the People's Republic of China. In our experiments, JNG and ATX doses were calculated based on human and animal equivalent dose formulas described in the second edition of Experimental Methodology of Pharmacology (1991).

### 2.3. Experimental Animals

Male SHRs (*n* = 50) and WKY rats (*n* = 10, aged 4 weeks) weighing 80–100 g were purchased from Beijing Vital River Laboratory Animal Technology Co. Ltd. (Beijing, China, Permission No. SCXK (J) 2016–0001). All animals were housed in an environment with a temperature of 22 ± 1°C, relative humidity of 50 ± 1%, and a light/dark cycle of 12/12 hr. All animal studies (including the rat euthanasia procedure) were conducted in accordance with the Animal Management Rules of the Chinese Ministry of Health and with the approval of the Animal Ethics Committee of Dongzhimen Hospital affiliated with Beijing University of Chinese Medicine (No. 21–27).

### 2.4. Experimental Design and Drug Treatment

The WKY rats served as control (*n* = 10). The SHRs were randomly divided into the following five groups (10 rats/group): model group (1 mL/100 g body weight [BW], distilled water); ATX (5.36 mg/kg BW, ATX) group; and low, medium, and high doses of JNG groups (5.785, 11.57, and 23.14 g/kg BW, JNG decoction). The corresponding treatments were administered to each group daily for 6 weeks. A behavioral test was carried out at the end of 6 week's treatment. After the behavioral test, all rats were individually killed by one experienced person by decapitation using a guillotine, and the brain tissue was quickly collected on ice for further investigation.

### 2.5. Behavioral Testing

#### 2.5.1. Step-Down Test

The apparatus consisted of a grid floor and a rubber platform. On a training day, a rat was located on the grid floor, and electrical stimulation (36 V) was delivered once it touched the grid floor. The normal response was to jump onto a rubber platform to avoid electrical stimulation. The learning and memory were assessed by measuring the step-down latency of the first step from the platform and the number of electric shocks received within 5 minutes (number of errors). If the rats did not jump off the platform within 5 min, the number of errors and the incubation period were recorded as 0 180 s, respectively.

#### 2.5.2. Open Field Test (OFT)

OFT was carried out from 9 : 00 to 17 : 00 in the daytime. The open field is a black box (100 × 100 × 40 cm) and for data analysis, the total area was equally divided into 16 equal squares virtually, a central area (50 × 50 cm), and a peripheral area (25 cm on each side). Each rat was gently placed in the center of the field and observed for 5 min. Arenas were cleaned with 75% isopropyl alcohol between trials to remove scent marks. Total exercise distance(cm), resting time (s), time spent in the central region (s), and time spent in edge area movement were recorded and analyzed by Easy-Tracking System (SLY-ETS Version 1.66, Beijing Sunny Instruments Co. Ltd.).

### 2.6. Histopathological Analysis

Brain tissue samples were fixed with 4% paraformaldehyde, embedded in paraffin, then cut into 6-*μ*m-thick sample sections. For hematoxylin/eosin-stained tissue samples, an SCN400 Digital Slide Scanner (Leica Microsystems, Wetzlar, Germany) was used to examine and acquire images to identify changes in neurons in the hippocampal CA1 area. Two independent examiners performed the diagnosis in a blinded manner.

### 2.7. ELISA for cAMP in the Striatum

The striatum was collected, and protein concentrations were measured by the ELISA kit (Reagent: Rat cAMP ELISA kit, Item No: E02C0027). The levels of cAMP in the striatum were detected following the manufacturer's instructions, and the sample concentration was determined using a standard curve.

### 2.8. Determination of Ca^2+^ Content in the Striatum

Samples were rinsed in ice-cold normal saline, dried using filter paper, weighed, and put in a 5-ml homogenization tube. Then, samples were mixed with a homogenization medium (pH 7.4, 0.01 mol/L Tris-HCl, 1 mmol/L EDTA-2Na, 0.01 mol/L sucrose, and 0.8% sodium chloride solution) to prepare 10% homogenate with a low-temperaturelow-speed centrifuge at 2500 rpm and centrifuged for 15 minutes. Finally, the supernatant was collected. The A045-4 total protein quantitative test kit (BCA Protein Assay method) was used to determine the protein concentration of the sample. Mtb:alkaline solution:protein clear solution = 10 : 20 : 1 was used to configure the working solution. Then, 10 ul of ionized water/hole and 10 ul of 1 mmol/L calcium standard solution/well were added to the standard and sample wells, respectively. This was followed by adding 10 ul/well of tissue homogenate supernatant and 250 ul/well of working solution, which was mixed well, and let stand for 5 minutes. Next, the OD value of each well was measured at 610 nm with a microplate reader.

### 2.9. Western Blot

The proteins were extracted from the striatum tissues using radioimmunoprecipitation assay (RIPA) lysis buffer (Ukzybiotech, Beijing, China) according to the manufacturer's instructions. The protein concentration in the lysates was evaluated using a BCA Protein Assay Kit. Proteins were then separated on an SDS-PAGE and transferred to a polyvinylidene difluoride membrane. Membranes were blocked with 5% BSA-TBST and then incubated with DA1R antibody (ab20066, 1 : 1000), DA2R antibody (ab85367, 1 : 1000), DA3R antibody (ab42114, 1 : 1000), DA4R antibody (ab20424, 1 : 500), DA5R antibody (ab40656, 1 : 200), PKA antibody (ab211265, 1 : 1000), CAM (ab45689, 1 : 1000), and CAMKII (ab52476, 1 : 1000) (Abcam) overnight at 4°C. Then, the blots were washed, incubated with 5% BSA-TBST, and washed again. Consequently, samples were incubated with horseradish peroxidase-conjugated secondary antibody at room temperature for 2 h. Band detection was performed using the enhanced chemiluminescence (ECL) detection kit. The intensity of the detected bands was calculated densitometrically using the Gel Image system ver.4.00 (Tanon, China).

### 2.10. RT-PCR

The total RNA of D1-like, D2-like, CaM, and CaMKII from the striatum or hippocampus samples was extracted using Trizol reagent according to the manufacturer's instructions. RNA was reverse transcribed to cDNA using a TIANScript RT Kit. cDNA was used as a template to perform PCR amplification with an SYBR FAST qPCR Kit Master Mix (2×) Universal (US KAPA Biosystems). The specific primers are shown in Table 2.

Each 20 *μ*L reaction consisted of 2 *μ*L of cDNA, 10 *μ*L of SYBR Master Mix (2×) Universal, and 10 *μ*mol/L of each primer (sense and antisense primers). Three replicates of each PCR run were performed. The mRNA concentrations of all target genes were normalized to that of GAPDH in each sample (using the delta-delta-Ct method).

### 2.11. Statistical Analysis

All data analyses were performed using the SPSS software version 19.0 (IBM Inc., Somers, NY, USA). Results were expressed as the mean ± standard deviation (SD). Statistical differences among these groups were evaluated using a one-way analysis of variance (ANOVA) followed by an LSD post hoc test. A *P* value < 0.05 was considered statistically significant.

## 3. Results

### 3.1. Step-Down Test

**Figure 2:**
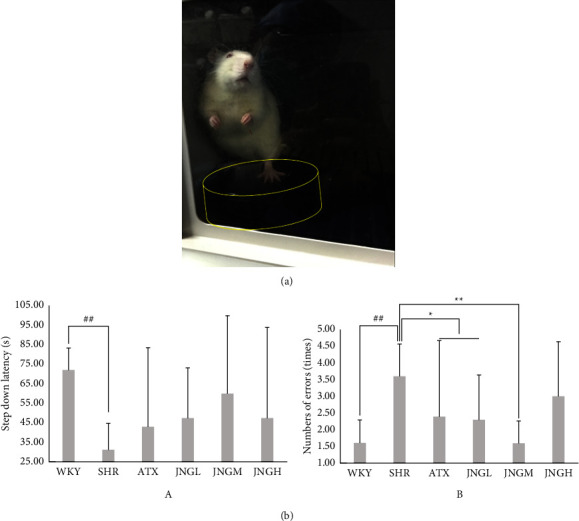
(a) Two independent trained observers assessed a randomized screenshot from a complete video recording showing the ADHD model rat standing on the platform. (b) The step-down latency and the number of errors after 6 weeks of treatment. Data are expressed as mean ± SD. ^##^*P* < 0.01 versus WKY group; ^*∗∗*^*P* < 0.01, ^*∗*^*P* < 0.05 versus SHR group.

As shown in Figure 2(b), the step-down latency was significantly shortened and the number of errors was significantly increased in the SHR group compared to the WKY group (*P* < 0.01). There was no difference in step-down latency between the ATX and JNG-treated groups (*P* > 0.05). JNG prolonged the step-down latency, especially in the JNGL group (Figure 2(b)(A)). Consistently, ATX, JNGM, and JNGL reduced the number of errors.

### 3.2. Open Field Test

**Figure 3:**
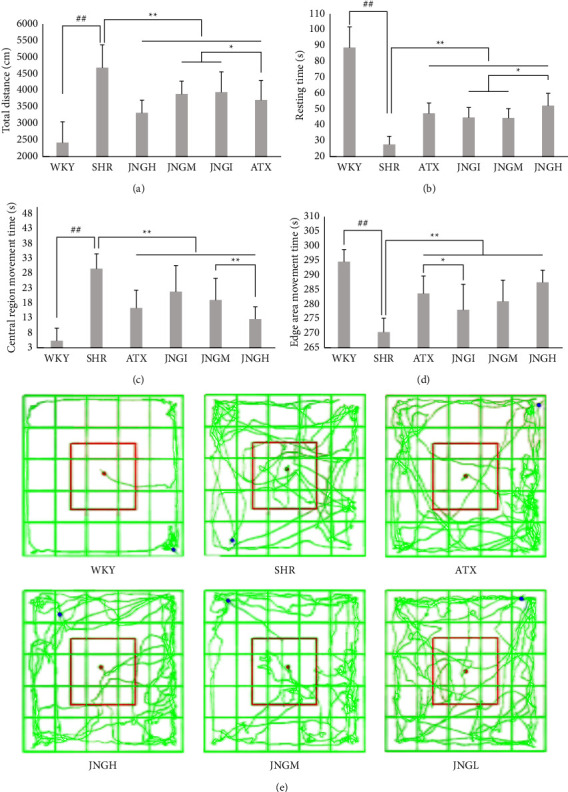
JNG ameliorates the hyperactive/impulsive behavior and cognitive function of ADHD rats. (a)–(d) Total distance, resting time, central area movement time, and edge area movement time in OFT (*n* = 10). (e) Representative trajectories of each group in OFT (*n* = 10).

As shown in Figure 3, the total exercise distance of the SHR group was significantly longer than that of the WKY group (*P* < 0.01). Moreover, the distance was shorter in the JNG and ATX groups vs. the SHR group (*P* < 0.01). The total exercise distance in the JNGH group was longer than that of the JNGM and JNGL groups (*P* < 0.05).

The resting time of rats in the SHR group was significantly shorter than that in the WKY group (*P* < 0.01). In addition, the resting time of each rat in the JNG and ATX groups was significantly increased compared to the SHR group (*P* < 0.01). Furthermore, the resting time of JNGH was significantly longer than that in the JNGM and JNGL groups (*P* < 0.05).

The central area exercise time of the SHR group was significantly longer than that of the WKY group (*P* < 0.01). The central time of the JNG and ATX groups was significantly reduced compared with the SHR group (*P* < 0.01). The central time of the JNGM was increased compared with the JNGH group (*P* < 0.01).

The movement time of the edge area of the SHRs was significantly lower than that of the WKYs (*P* < 0.01). On the other hand, the movement time of the JNG and ATX groups was significantly increased than that of the SHR group (*P* < 0.01). Additionally, the movement time of the edge area of the JNGL group was lower than that in the ATX group (*P* < 0.05).

The open field motion trajectory diagrams of each group of rats were analyzed. The rats in WKYs had a single clear motion route along the edge of the box mainly, and the overall motion path was short, while rats in the SHR group had complex, chaotic motion routes and crossed the central area many times instead of routes parallel to the edge of the box. Rats in the other groups ran away from the central area and increased the movement time in the edge area, so the route was more regular when compared with that in the model group.

### 3.3. Hippocampal CA1 Area Histomorphological Changes

**Figure 4:**
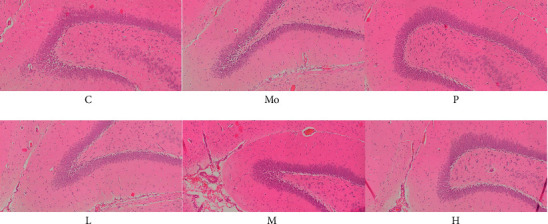
Brain sections for histology were stained with hematoxylin and eosin. Representative photomicrographs are shown for each group (HE, ×10). C: WKY group, Mo: model group (SHR), P: ATX group, L: JNGL group, M: JNGM group, H: JNGH group.

As shown in Figure 4, the number of neurons in the hippocampal CA1 area of the SHR group was significantly reduced compared with the WKY group, the nerve cells were unevenly and loosely arranged, and the boundary was blurred. Conversely, the number of neurons in the hippocampal CA1 area of the JNG group and the ATX group was increased compared with the model group; also, the arrangement was neater, and the intercellular space was reduced. In addition, these changes were particularly evident in the JNGH group.

### 3.4. The Expression of Dl-Like (D1 + D5) and D2-Like (D2 + D3 + D4) Proteins and the D2-Like/Dl-Like Ratio in the Striatum

**Figure 5:**
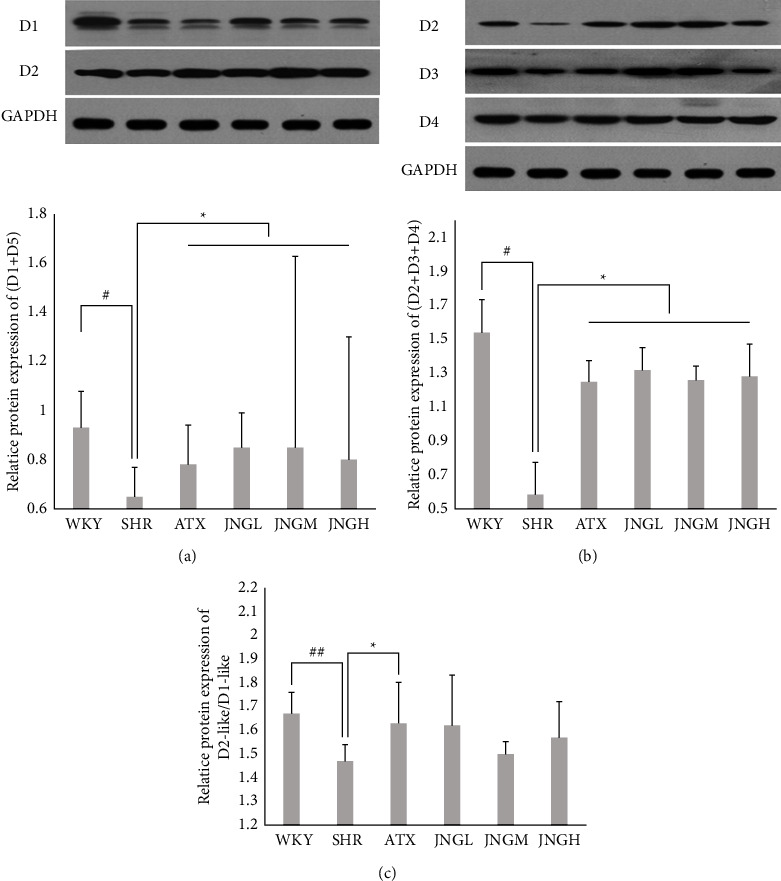
(a) Representative western blot analysis of the expressions of D1 and D5 in the striatum samples after 6 weeks of treatment. (b) Representative western blot analysis of the expressions of D2, D3, and D4 in the striatum samples. (c) The protein ratio of D2-like/Dl-like in the striatum after 6 weeks of treatment. Data are expressed as mean ± SD. ^##^*P* < 0.01, ^#^*P* > 0.05 versus control group; ^*∗*^*P* > 0.05 versus model group.

Western blot was used to determine whether or not medications could regulate the protein expression of Dl-like (Figure 5(a)), D2-like (Figure 5(b)), and the ratio of D2-like/Dl-like (Figure 5(c)) in the striatum. The expression of Dl-like and D2-like patients and the ratio of D2-like/Dl-like in the SHR group were decreased compared to the WKY group (*P* < 0.05 for Dl-like and D2-like;*P* < 0.01 for D2-like/Dl-like). Moreover, ATX and JNG both increased the protein levels of Dl-like and D2-like in the striatum when compared with the SHR group (*P* < 0.05); yet, JNG could not affect the ratio of D2-like/Dl-like proteins (*P* > 0.05), while an upregulated D2-like/Dl-like protein level was observed in the ATX group when compared to that in the SHR group (*P* < 0.05).

### 3.5. The Expression of Dl-like (D1 + D5) and D2-Like (D2 + D3 + D4) mRNA and the D2-Like/Dl-Like mRNA Ratio in the Striatum

**Figure 6:**
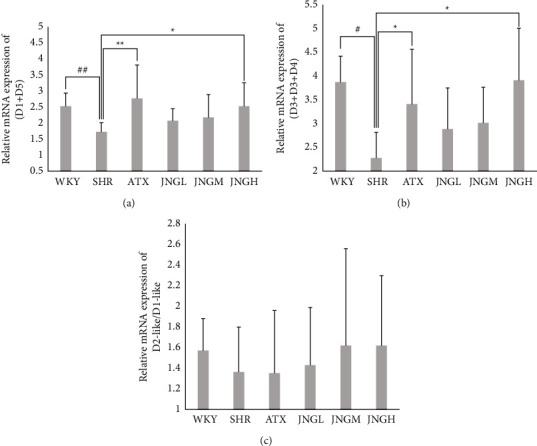
(a)–(c) The mRNA expression of Dl-like (D1 + D5) (a), D2-like (D2 + D3 + D4) (b), and D2-like/Dl-like (c) mRNA ratio in the striatum after 6 weeks of treatment. Data are expressed as mean ± SD. ^##^*P* < 0.01, ^#^*P* > 0.05 versus WKY group; ^*∗∗*^*P* < 0.01, ^*∗*^*P* > 0.05 versus SHR group.

The Dl-like and D2-like mRNA levels and the ratio of D2-like/Dl-like in the striatum of rats in each group are shown in Figures 6(a)–6(c). The mRNA expression of Dl-like and D2-like in the SHRs showed a significant decrease compared with the WKY group. The mRNA expression of Dl-like and D2-like in the ATX and JNGH groups was upregulated, but there was no effect on the D2-like/Dl-like mRNA ratio. The data showed an upward ratio trend in JNG group, especially in the JNGM and JNGH, when compared with the SHRs.

### 3.6. cAMP Levels in the Striatum

**Figure 7:**
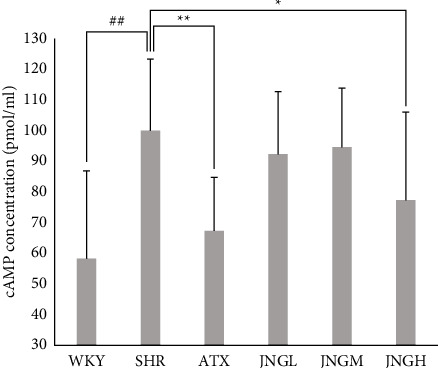
cAMP levels in the striatum after 6 weeks of treatment. Data are expressed as mean ± SD, ^##^*P* < 0.01 versus WKY + distilled water group (control group); ^*∗∗*^*P* < 0.01, ^*∗*^*P* > 0.05 versus SHR + distilled water group (model group).

As shown in Figure 7, quantitative results revealed that cAMP concentrations in the striatum in the SHR group were significantly increased compared with the WKY group (*P* < 0.01), while the cAMP levels in the striatum of ATX and high dose of JNG groups decreased significantly (all *P* < 0.05).

### 3.7. The Protein Expression of PKA in the Striatum

**Figure 8:**
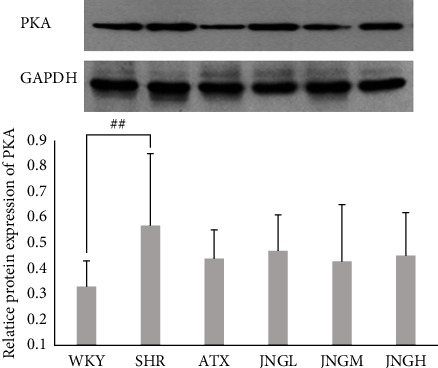
The protein expression of PKA in the striatum after 6 weeks of treatment. Representative western blot in PKA for the striatum samples. Data are expressed as mean ± SEM. *n* = 10, ^##^*P* < 0.01 versus WKY + distilled water group (control group).

To further investigate the activity and amount of PKA in the striatum of rats treated with various medications, PKA was assessed by western blot. As shown in Figure 8, the expression of PKA in the striatum of the SHR group increased compared with that in the WKY group (*P* < 0.01). However, ATX and JNG showed low effects on the protein expression of PKA when compared with SHRs; the levels of PKA increased the most in the JNGM group.

### 3.8. The Content of Ca^2+^ in the Striatum

**Figure 9:**
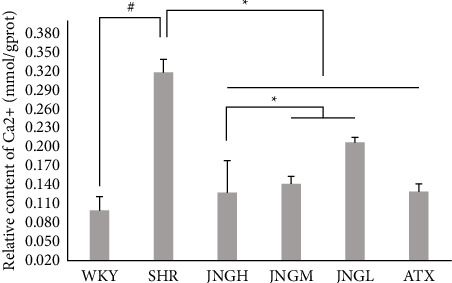
The calcium content in the striatum of rats from each group after 6 weeks of treatment. Data are expressed as mean ± SD. ^#^*P* < 0.05 versus WKY + distilled water group (control group); ^*∗*^*P* < 0.05 versus SHR + distilled water group (model group).

Figure 9 shows that the calcium content of the striatum of rats from the model group was significantly higher than that of the WKY group (*P* < 0.05). Compared with the SHRs, the calcium content in the striatum of rats from the ATX and JNGs was downregulated (*P* < 0.05).

### 3.9. The Expression of CaM and CaMKII Proteins in the Hippocampus

**Figure 10:**
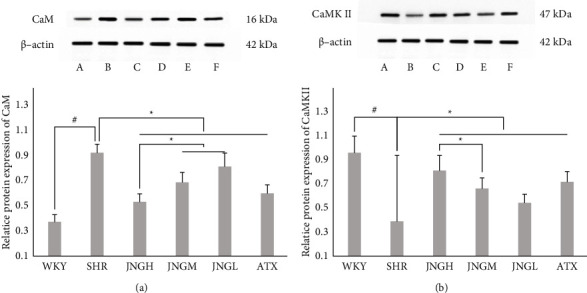
(a) Representative protein expression of CAM and western blot in the hippocampus of rats from each group after 6 weeks of treatment. (b) Representative protein expression of CAMKII and western blot in the hippocampus after 6 weeks of treatment. Data are expressed as mean ± SD. ^#^*P* < 0.05 versus WKY + distilled water group (control group); ^*∗*^*P* < 0.05 versus SHR + distilled water group (model group).

As shown in Figure 10, CaM expression in the hippocampal area of rats in the model group was significantly higher than that of the WKY group (*P* < 0.001), while the CaM levels in the ATX, JNGM, and JNGH groups were lower compared with the SHR group.

CaMKII protein in the hippocampus of rats of the SHR group was significantly lower than that in the WKY group. Compared with the model group, CaMKII protein in the hippocampus from the ATX group and the JNGM and JNGH was significantly upregulated; however, there was no difference between the JNGM and JNGH groups (*P* > 0.05).

### 3.10. The mRNA Expression of CaM and CaMKII in the Hippocampus

**Figure 11:**
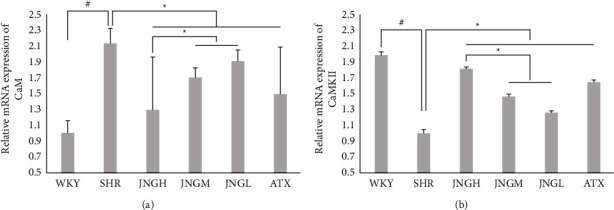
(a), (b)The mRNA expression of CaM (a) and CaMKII (b) in the hippocampus after 6 weeks of treatment. Data are expressed as mean ± SEM. *n* = 7, ^*∗*^*P* < 0.05 versus WKY + distilled water group (control group); ^#^*P* < 0.05 versus SHR + distilled water group (model group).

As shown in Figure 11, the mRNA expression of CaM in the hippocampus of rats in the SHR group was significantly higher than that of the WKY group (*P* < 0.05). The mRNA of CaM in the ATX and the JNGH was significantly reduced compared to the SHR group.

The mRNA expression of CaMKII in the hippocampus in the SHRs was significantly lower than that of the WKYs (*P* < 0.05). However, after treatment, the hippocampal CaMKII mRNA expression in the ATX group and the JNGs were significantly increased compared with the SHR group (*P* < 0.05).

## 4. Discussion

Our results revealed a significant imbalance in D2/D1 receptors and upregulation of the cAMP/PKA and Ca^2+^/CaM/CaMKII signaling pathways in the ADHD rat model. Moreover, JNG could improve core symptoms and regulate the balance of D2/D1 receptors and their mediated cAMP/PKA and Ca^2+^/CaM/CaMKII signaling pathways.

ADHD is a chronic psychiatric neurological disorder with unclear etiology. Long-term drugs are commonly used to control core symptoms [35, 36]. Central stimulants and noncentral stimulants are considered first-line drugs in clinic. However, these treatments are also associated with certain side effects, including headache, insomnia, loss of appetite, abdominal pain, and twitching. They can also lead to drug abuse, negatively affect growth and development, and lead to depression [23, 37].

TCM has unique guiding principles and characteristics in diagnosis and treatment. Further research on TCM can provide a feasible way to prevent and treat ADHD. “Suwen (The Plain Questions)-Shengqi-Tongtianlun” states: “Yang is active, and Yin is static. When Yin and Yang are balanced, the human body can coordinate its movement and staticity.” Our research group combined the theory of “Yin-Yang imbalance” with the physiological characteristics of children's “delicate viscera and insufficient body Qi” where “Yang is always in excess, while Yin is always insufficient,” thus creating the prescription of JNG for the treatment of ADHD on the basis of many years of clinical practice. JNG can replenish Qi and nourish Yin, calm the mind, and benefit the intellect, therefore restoring the homeostasis of the body's dynamic balance of Yin and Yang. UPLC-MS/MS investigated the quality control of JNG, and the major photochemical constituents of JNG extract have been identified [28]. Previous studies suggested that JNG could significantly improve hyperactivity, inattention, learning and memory, cognitive functions, etc., without obvious adverse reactions. In this study, we verified the efficacy of JNG therapy on a rat model of ADHD and discussed its mechanism by regulating the balance of D2/D1 receptors and their downstream signaling pathways. This research provides a new perspective and theoretical basis for the development of new drugs for treating ADHD.

In this study, SHRs were used as a model of ADHD. SHR is an animal model isolated by selective inbreeding of WKY rats. Compared with WKY rats, juvenile SHR shows behavioral characteristics such as spontaneous hyperactivity, attention deficit, and impulsivity. It has been proposed that prepubertal 4-week-old SHRs develop behavior characteristic of ADHD without hypertension [38]. Preclinical studies have shown that SHRs have good apparent, structural, and predictive validity, which enable the assessment and development of new therapeutic interventions [39].

Dopaminergic neurons project to several brain regions, such as the prefrontal cortex (PFC), striatum, and hippocampus, and participate in high-level neural functions such as attention allocation, motor control, learning, and memory [40–42]. Distinct structural and functional cerebral abnormalities in the PFC, hippocampus, and striatum have been found in patients with ADHD [43, 44]. The PFC and striatum have an important role in attention regulation, hyperactivity impulsivity, working memory, and emotional control [45]. The hippocampus is an important organ in the central nervous system that participates in learning and memory storage [46]. These areas are interconnected by a network of neurons and regulate attention, thoughts, emotions, behavior, and actions. Thus, it is of practical significance to study the related indexes of the striatum and hippocampus in the brain tissue of the ADHD model.

In this study, the step-down test and OFT were used to examine the effect of JNG on the ADHD rat model. The step-down test is a relatively simple experiment used to study learning, memory, and cognitive function [47]; the OFT is a common experimental method to evaluate the autonomous movement ability of animals. The distance and speed of the rats in the open field experiment are often used to evaluate the two core symptoms of ADHD, that is, hyperactivity and impulsivity [48]. In this study, these behavioral studies revealed typical characteristics of ADHD hyperactivity, attention deficit, learning and memory, and cognitive impairment in SHR rats, which are in line with ADHD's clinical manifestations. In contrast, the intervention of JNG can improve ADHD's hyperactivity, inattention, learning, memory, and cognitive dysfunction and control the core symptoms of ADHD, which is consistent with previous studies [31–33].

At present, the pathogenesis and location of ADHD are not clear. Moreover, as the basic unit of function and structure of the nervous system, there are few studies on the effects of drugs on neurons. In this experiment, the mechanism of TCM treatment of ADHD was explored from the perspective of neuronal pathology, and the hippocampus was used as the sensitive area of ADHD to study the pathological changes in the hippocampus during the treatment of ADHD [49]. Hematoxylin and eosin (H&E) staining showed that the tissue structure of the hippocampus and surrounding areas tended to be normal in rats treated with JNG, and the number and morphology of neurons in the CA1 area were significantly improved. This study suggests that JNG has protective and repair effects on neurons in ADHD rats, which lays a direction for the next step, that is, to determine the research target and the mechanism of neuronal repair.

Dysfunction of dopaminergic neurons is one of the main causes of neuropsychiatric diseases related to the onset of ADHD [49]. Previous neurobiochemical experiments confirmed that JNG could increase the DA content in the brain tissue of SHR rats [31–33]. It is widely recognized that DA receptor genes are the most important components in the etiology of ADHD among many candidate genes [3]. According to their different biochemical, pharmacological, and physiological attributions, DA receptors can be divided into two distinct subtypes: the D1-like receptor family, including dopamine D1 and D5 receptors, and the D2-like receptor family, which includes dopamine D2, D3, and D4 receptors [14, 15]. As receivers of DA signals, they have an important role in the feedback regulation of the physiological effects of DA and its content level and can perform all physiological functions of dopamine. Previous studies have confirmed the critical involvement of an imbalance between the levels of D1-like and D2-like receptors in many central nervous system diseases [50, 51]. Our results (Figure 5(c)) showed that the ratio of D2-like/D1-like is greater than 1, which suggests that D2-like receptors are more expressed than D1-like receptors. This also means that the D2-like receptors have a dominant role in the DA receptor family. Comparing the model group with the WKY group, it was found that the ratio of D2-like/D1-like in the model group was notably lower than that of the WKY group, but it was increased following the treatment with ATX and JNG. This revealed that the D1-like receptors of the SHRs are relatively more prominent than that of the WKY group, and this excitatory action may be corrected by drug treatment. From the perspective of TCM, the physiological effects of excitability produced by D1-like receptors can be attributed to Yang hyperactivity. Normally, D1 and D2-like receptors balance each other, like Yin and Yang balance each other and are interdependent. JNG adjusts the imbalance by alleviating the hyperfunction of D1-like receptors.

The executive function of the DA receptor is closely related to its downstream signaling pathways that are crucial to maintaining physiological processes. An unbalanced activity may lead to dysfunction related to the onset and progression of ADHD. The cAMP/PKA and Ca^2+^/CaM/CaMKII signaling pathways have an important role in intracellular signal transduction and are essential in mediating DA receptors to perform their physiological functions (such as voluntary movement, working memory, attention, and learning). Based on the abovementioned results, we targeted cAMP/PKA and Ca^2+^/CaM/CaMKII signaling pathways [52] to further elucidate the molecular mechanism underlying action in the ADHD model.

It is commonly accepted that D1-like receptors are coupled to G*α*s/olf proteins, activating the adenylyl cyclase to produce higher levels of the second messenger cAMP, which stimulates the activity of the protein kinase A (PKA). In contrast, D2-like receptors, which are coupled to G*α*i/o protein, inhibit adenylyl cyclase and reduce the intracellular concentration of cAMP that, in turn, blocks PKA activity [53]. Therefore, the activity and expression level of cAMP/PKA is controlled by D1 and D2-like receptors. cAMP is an important intracellular second messenger that can enhance, differentiate, and integrate the information obtained and transmit it to the corresponding effectors to perform specific physiological functions. For example, it regulates some normal brain functions, including neuronal memory and synaptic plasticity, which are related to ADHD [54]. PKA is a key regulator of cAMP that can phosphorylate a series of substrates. The activation of PKA participates in multiple types of cognitive performance, synaptic plasticity, and long-term memory and affects human cognition [55]. It has been proved that the degree of PKA activation affects the cognitive level of the human body. Abnormal PKA levels can inhibit the expression of cAMP/PKA/cAMP-response element binding protein (CREB)-related factors, leading to attention deficits and hyperactivity [56].

The Ca^2+^/CaM/CaMKII signaling pathway is another regulatory pathway associated with ADHD. Ca^2+^ homeostasis imbalance may lead to the pathological value of ADHD, which has been gradually recognized by the medical community and has become the research focus [57]. In the process of cell signal transduction, CaM is a receptor protein of Ca^2+^. Most of the signal transduction mediated by Ca^2+^ goes through the Ca^2+^/CaM-dependent protein kinase pathway, which has many physiological functions such as regulating the synthesis of neurotransmitters, synaptic plasticity, learning, and memory. CaMKII is an important target enzyme of Ca^2+^. It is a silk/threonine protein kinase mainly expressed in the hippocampus that has the highest content in brain tissue and can be regulated by autophosphorylation. When the intracellular Ca^2+^ concentration increases, the interaction between Ca^2+^/CaM and CaM causes a conformational change, inactivates the self-inhibitory region, and transmits Ca^2+^ signals through the phosphorylation of many substrate proteins [58, 59]. CaMKII can cause CREB phosphorylation, thereby regulating gene transcription in the hippocampus and enhancing the formation of long-term potentiation (LTP) in the hippocampus through the expression of downstream genes [60]. Due to the antagonistic effects of D1 excitability and D2 inhibition, the excitability or inhibition of the Ca^2+^/CaM/CaMKII signaling pathway differs depending on the dominant position of different receptors. It regulates synaptic plasticity by inducing LTP and long-term inhibition (LTD) related to learning, memory, and cognition [61, 62]. Taken together, the cAMP/PKA and Ca^2+^/CaM/CaMKII signaling pathways represent an important target for ADHD.

In this study, our results (Figures 7 and 8) showed significant upregulation of key factors in the model group's cAMP/PKA signaling pathway. This abnormal expression suggested that the imbalance between D1 and D2-like receptors in the model group might be associated with increased activation of the cAMP/PKA signaling pathway. JNG's ability to downregulate the expression of key molecules of the cAMP/PKA signaling pathway indicated that this drug has inhibitory effects on the pathway. When the excitatory D1-like receptors are dominant and relatively hyperactive, the Ca^2+^/CaM/CaMKII signal pathway in SHRs is abnormally excited. JNG can significantly downregulate the protein and mRNA level of Ca^2+^/CaM (Figures 9 and 10) and upregulate the protein and mRNA expression of CaMKII (Figure 11). Therefore, the underlying mechanism of JNG treating ADHD might be involved in modulating the Ca^2+^/CaM/CaMKII signaling pathway.

Our preliminary results highlight the pathogenesis of ADHD by focusing on dopaminergic D2/D1-like receptors and related cAMP/PKA and Ca^2+^/CaM/CaMKII pathways. Studying more than one downstream signal pathway of DA receptors is more informative rather than studying just a single signal pathway, as it extends the research objective from analyzing single factors to analyzing overall coordination. Furthermore, to the best of our knowledge, this is the first study that used dopaminergic D2/D1-like receptor-mediated signaling pathways as the focus of research on the mechanism of action of JNG. Our results revealed that the underlying mechanism of JNG alleviates ADHD in rats by modulating dopaminergic D2/D1-like receptor-mediated signaling pathways, enriches the scientific connotation of JNG in treating ADHD, and provides new research ideas for the treatment of ADHD with TCM.

This study has some limitations. First, JNG is a compound preparation comprising a variety of traditional Chinese medicinal components; thus, the single component role in JNG treatment needs to be further explored. Second, we did not consider inhibitors of the cAMP/PKA and Ca^2+^/CaM/CaMKII signaling pathways. Finally, we did not test the JNG effects using cell experiments. Therefore, future studies should use various inhibitors and detection methods to validate the exact regulatory targets and underlying mechanisms of JNG.

## 5. Conclusion

This study suggests that JNG may be used to treat ADHD. The underlying mechanism of the neuroprotection effect might be involved in regulating the balance of the dopaminergic D2/D1-like receptors. Moreover, our data showed that JNG could coordinate D2/D1 balance and D2/D1-like receptor-mediated-cAMP/PKA and Ca^2+^/CaM/CaMKII signaling pathways, improving attention deficit hyperactivity disorder, impulsive behavior, learning, memory, and cognitive functions. These findings provide a new valuable insight into the pathogenesis and therapeutic target of ADHD with TCM.

## Figures and Tables

**Table 1:** Components of the JNG.

Full botanical plant name	Chinese name	Part used	Weight (g)
*Pseudostellaria heterophylla* (Miq.)	Taizishen	Root	12
*Rehmannia glutinosa* (Gaertn.) DC.	Shudihuang	Root	12
*Schisandra chinensis* (Turcz.) Baill.	Wuweizi	Mature fructus	6
*Lycium barbarum* L.	Gouqizi	Mature fructus	6
*Poria cocos* (Schw.) Wolf	Fuling	Sclerotium	12
*Polygala tenuifolia* Willd	Yuanzhi	Root	6
*Acorus tatarinowii* Schott	Shichangpu	Rhizoma	6

*Note.* The plant names have been checked on https://www.theplantlist.org, mentioning the website's date.

**Table 2:** Specific gene primers.

Gene	Forward primer	Reverse primer
DAR1	GTGGGCGAATTCTTCCCTGA	GGGCAGAGTCTGTAGCATCC
DAR2	TAGGTACTGGCACCGGACTT	GCCAGGCTCACGATGAAGTA
DAR3	CACTCGACAGAACAGCCAGT	GGCTGCAGGTGTGACAAAAG
DAR4	TGGGCCAGAGTCCTTAGGTA	GAGTTCCCTGCCAACACCAT
DAR5	CCAAGACACGGTCTTCCACA	CTGGCGTCGTTGGAGAGATT
CAM	TACGCTAGGCCAATATACAA	TACGCTAGGCCAATATACAA
CAMKII	AAGATGTGCGACCCTGGAATG	TGTAGGCGATGCAGGCTGAC
GAPDH	TGTGAACGGATTTGGCCGTA	TGTGAACGGATTTGGCCGTA

## Data Availability

The data that support the findings of this study can be obtained from the corresponding author upon request.

## Abbreviations

ADHD: Attention deficit hyperactivity disorder
JNG: Jing-Ning granules
TCM: Traditional Chinese medicine
SHR: Spontaneously hypertensive rat
WKY: Wistar-Kyoto
ATX: Atomoxetine
OFT: Open field test
RT-PCR: Real-time polymerase chain reaction
cAMP: Cyclic adenosine monophosphate
ELISA: Enzyme-linked immunosorbent assay
ASD: Autism spectrum disorder
DA: Dopamine
NE: Norepinephrine
5-TH: 5-Hydroxytryptamine
DAT: Dopamine transporters
MAO: Inactivating enzymes
AC: Adenylate cyclase
H & E: Hematoxylin and eosin
CREB: cAMP-response element binding protein
LTP: Long-term potentiation
LTD: Long-term inhibition.
